# Determination of Post-Fermentation Waste from Fermented Vegetables as Potential Substitutes for Preservatives in o/w Emulsion

**DOI:** 10.3390/ijms25105510

**Published:** 2024-05-18

**Authors:** Anna Herman, Olga Matulewicz, Eliza Korzeniowska, Andrzej Przemysław Herman

**Affiliations:** 1 Faculty of Chemistry, Warsaw University of Technology, Koszykowa 75 Street, 00-662 Warsaw, Poland; 2 Department of Genetic Engineering, The Kielanowski Institute of Animal Physiology and Nutrition, Polish Academy of Sciences, Instytucka 3 Street, 05-110 Jabłonna, Poland

**Keywords:** fermented vegetable extract, antimicrobial activity, antioxidant activity, preservative efficiency

## Abstract

Post-fermentation wastes are rich sources of various biologically active compounds with antimicrobial activity, whose potential is not being fully exploited. One of the possible applications of post-fermentation waste may be its use as a natural preservative that effectively combats pathogens found in formulations. The study aims included the following: (1) compare the antimicrobial and antioxidant activity of fermented vegetable extracts (FVEs), (2) examine the inhibition of cosmetic-borne pathogens by FVEs, and (3) estimate the preservative effectiveness of FVEs in o/w emulsions. It was found that fermented white cabbage, cucumber, celery, and the mixture of fermented white cabbage, cucumber, and celery (1:1:1) showed antibacterial and antifungal activity against all the tested reference microbial strains. The addition of fermented cucumber, celery, and the mixture of fermented white cabbage, cucumber, and celery (1:1:1) to the o/w emulsion fulfilled criterion A of the preservative effectiveness test for *S. aureus*, *E. coli*, and *A. brasiliensis*, but did not fulfill the criterion for *P. aeruginosa* and *C. albicans*. The tested FVEs have comparable activity to inhibit pathogens in o/w emulsion as sodium benzoate. The results of our study prove that FVEs can be valuable raw materials supporting the preservative system, which, in turn, can significantly reduce the concentration of preservatives used in o/w emulsion.

## 1. Introduction

Food fermentation is regarded as one of the oldest ways of food processing and preservation. Fermentation enhances the flavor and nutritional quality of food and makes it a rich source of beneficial ingredients for human health (vitamins, probiotics, antioxidants, minerals). Therefore, fermented food, especially the fermented vegetables industry is the fastest-growing segment of the agricultural production market in the world. Unfortunately, while fermented vegetables are a valuable and valued source of food, the post-fermentation wort is a waste product of the food industry, whose potential is not being fully exploited.

Fermented vegetable extracts (FVEs) are raw materials obtained by the fermentation of vegetables with lactic acid bacteria (LAB). LAB is a group of Gram-positive, catalase-negative, microaerophilic, non-sporing cocci, coccobacilli, or rods with lactic acid as the main product of carbohydrate fermentation [1]. Most LAB (*Lactobacillus*, *Pediococcus*, *Enterococcus*, *Leuconostoc*) inhibit pathogenic, non-pathogenic, and spoilage organisms in fermenting foods and beverages [2]. The antimicrobial activity of LAB strains is attributed to the production of metabolites such as organic acid (lactic, acetic, formic, propionic acids), diacetyl, hydrogen peroxide, bacteriocins (nisin, reuterin, pediocin, lacticin, enterocin), antifungal compounds (propionate, phenyl-lactate, hydroxyphenyl-lactate, cyclic dipeptides, and 3-hydroxy fatty acids) and antimicrobial peptides causing non-specific inhibition of pathogen development [3,4]. Lactic and acetic acids effectively inhibit the growth of bacteria, mold, and yeast [3,5,6]. Their mechanism of action involves lowering the pH of the environment to a level unfavorable for pathogens (below pH 4), as well as disturbing the metabolic processes taking place in microbial cells and the active transport through cell membranes. Diacetyl is more effective against Gram-negative bacteria, yeasts, and molds than against Gram-positive bacteria by inactivating the arginine metabolic pathway [7]. Hydrogen peroxide has strong antimicrobial activity, consisting of the denaturation of cellular enzymes and peroxidation of membrane lipids, which lead to disruption of cell membrane function and the stopping of many metabolic pathways [3]. Bacteriocins are ribosomally produced antimicrobial peptides by LAB, either processed or not by additional posttranslational modification enzymes and exported to the extracellular medium [8]. Bacteriocins may have a bacteriostatic or bactericidal activity on closely related microbial species [9]. Most of them damage the cell membrane of microorganisms and disturb the internal metabolism of their cells [10]. Bacteriocins from LAB are GRAS (Generally Recognised As Safe) compounds that continue to gain interest due to their potential as safe food preservatives [11], alternatives for medically important antibiotics for treating infectious diseases [12], and substitutes for cosmetic preservative systems [13]. Therefore, fermented plants with antimicrobial activities could be a valuable raw material for the cosmetics industry. Moreover, fermented plants have many other valuable properties, such as antioxidant, anti-inflammatory, anti-melanogenic, and wound-healing activity useful for the cosmetics industry [14].

Industrial food waste may have the potential to generate income from antimicrobial compounds obtained through fermentation as valuable raw materials for the pharmaceutical and cosmetics industry. One of the possible applications of post-fermentation waste may be its use as a natural preservative that effectively combats pathogens found in formulations. The study aims include the following: (1) compare the antimicrobial and antioxidant activity of FVEs, (2) examine the inhibition of cosmetic-borne pathogen growth by FVEs and their mixtures, and (3) estimate the preservative effectiveness of FVEs and their mixtures in o/w emulsions.

## 2. Results

### 2.1. Number of LAB in FVEs

All FVEs contained both *Lactobacillus* and *Leuconostoc* strains (Table 1). None of the FVEs contained *Bifidobacterium* sp. Among the FVEs, the richest source of *Lactobacillus* sp. and *Leuconostoc* sp. were the fermented garlic (1 × 10^8^ cfu/mL) and fermented carrot (1 × 10^9^ cfu/mL), respectively. The smallest amount of *Lactobacillus* sp. and *Leuconostoc* sp. were observed in the fermented beetroot with 1 × 10^4^ cfu/mL and 5 × 10^3^ cfu/mL, respectively. None of the FVEs contained *Bifidobacterium* sp. 

Moreover, the presence of LAB and organic acid produced by these microorganisms lowered the pH of the FVEs to acidic values. All the bioferments before and after lyophilization had an acidic pH ranging from 3.42 (fermented carrot) to 4.44 (fermented cauliflower) for non-lyophilized samples, and from 2.9 (fermented cucumber) to 3.91 (fermented white cabbage) for the lyophilized samples (Table 2).

### 2.2. Antioxidant Activity of FVEs

**Figure 1:**
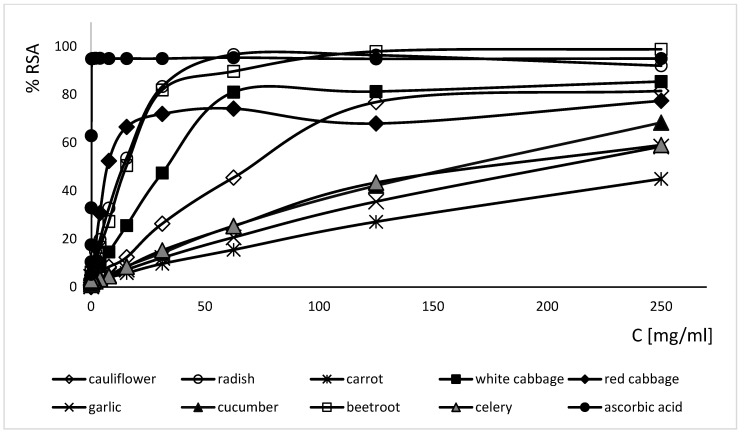
Radical scavenging activity (RSA) of fermented vegetable extracts [%].

All tested FVEs show antioxidant activity (Figure 1). EC50 estimation of the antioxidant activity of the FVEs showed that fermented red cabbage has the most effective concentration of antioxidant compounds that results in 50% inhibition of radical formation (EC50 = 7.14 mg/mL), while the fermented carrot has the weakest antioxidant activity (EC50 = 265.16 mg/mL) (Table 3). The antioxidant activity of fermented red cabbage was weaker compared to the antioxidant activity of ascorbic acid (EC50 = 0.1 mg/mL).

### 2.3. Antimicrobial Activity of FVEs and Their Mixtures

The antimicrobial activity of the FVEs and their mixtures were checked in the diffusion test and are presented in Table 4. The fermented white cabbage, fermented cucumber, and fermented celery showed the strongest antibacterial activity against *S. aureus*, *P. aeruginosa*, and *E. coli* growth. Moreover, fermented white cabbage and fermented cucumber inhibited *C. albicans* growth, while fermented celery showed antifungal activity against *A. brasiliensis*. Fermented carrot, fermented red cabbage, and fermented garlic showed antibacterial activity only against *P. aeruginosa*, and no antifungal activity was observed. Fermented radish and fermented beetroot did not inhibit the growth of bacteria, yeast, and mold. The mixture of fermented white cabbage, fermented cucumber, and fermented celery (1:1:1) showed the strongest antimicrobial activity compared to all other mixtures and inhibited all the bacteria and yeast tested. Therefore, the fermented white cabbage, fermented cucumber, fermented celery, and their mixture (1:1:1) were selected for further study.

### 2.4. The Microbiological Purity of the o/w Emulsions

All prepared o/w emulsions (base, preservative, FVEs, and their mixtures) fulfilled the quantitative and qualitative conditions of microbiological purity for cosmetics according to EN ISO standards (Table 5). The total aerobic mesophilic microorganisms for all tested o/w emulsions were below 100 cfu/g and fulfilled the criterion for cosmetics category 1 (cosmetics for children; in the eye area and mucous membranes). Moreover, yeast and mold were not detected in the o/w emulsions. Among the microbial colonies, no pathogenic bacteria (*S. aureus*, *P. aeruginosa*, *E. coli*) or yeast (*C. albicans*) were detected.

### 2.5. Antimicrobial Effectiveness Test

**Figure 2:**
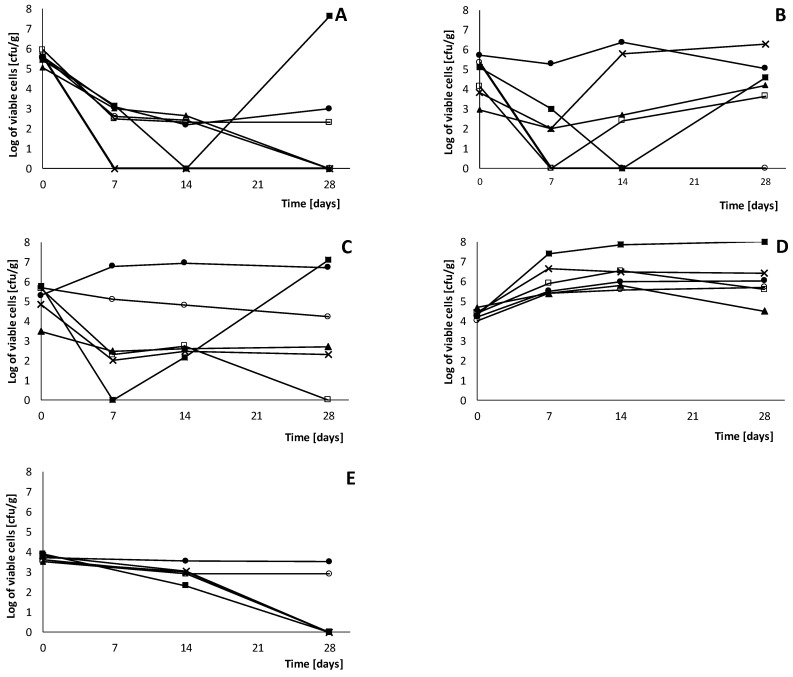
Inhibition of growth of *Staphylococcus aureus* ATCC 6538 (**A**), *Pseudomonas aeruginosa* ATCC 9027 (**B**), *Escherichia coli* ATCC 8739 (**C**), *Candida albicans* ATCC 10231 (**D**), *Aspergillus brasiliensis* ATCC 16404 (**E**) in o/w emulsion (Base) (●), o/w emulsion with preservative sodium benzoate (○), o/w emulsion with fermented white cabbage (■), fermented cucumber (□), fermented celery (▲), and mixtures of fermented white cabbage, cucumber and celery (1:1:1) (**X**).

The FVEs that were added to the o/w emulsion at a concentration of 6% and the synthetic preservative (0.5% sodium benzoate) protect the o/w emulsion against the growth of Gram-positive bacteria but are less effective against Gram-negative bacteria (Figure 2). 

Among all tested bioferments, fermented celery and a mixture of fermented white cabbage, cucumber, and celery (1:1:1) completely inhibited *S. aureus* growth in o/w emulsion after 28 days of incubation, while the fermented cucumber significantly inhibited the growth of *E. coli*. The synthetic preservative completely inhibited *P. aeruginosa* and *S. aureus* growth in the o/w emulsion after 28 days of incubation. Moreover, antimicrobial activity of fermented cucumber, celery, and the mixture of white cabbage, cucumber, and celery (1:1:1) in o/w emulsion were comparable with the activity of the synthetic preservative against *S. aureus, A. brasiliensis*, and both were not effective against *C. albicans*. The fermented cucumber, celery, and mixture of white cabbage, cucumber, and celery (1:1:1) inhibited *E. coli* growth, while the synthetic preservative was not effective against *E. coli*. In turn, the synthetic preservatives inhibited *P. aeruginosa* growth, while all the fermented vegetable extracts were not effective. The fermented vegetable extracts added to the o/w emulsion effectively reduced the growth of *A. brasiliensis*. None of the tested bioferments inhibited yeast *C. albicans* growth. These results were comparable with the results obtained for the synthetic preservatives, which also inhibited the growth of mold but not yeast.

According to the PN-EN ISO 11930:2019-03/A1:2023-02 standard [21], a preservative system meets the requirements when the following observations are made in the formulation: (1) a significant decrease in the number of viable microorganisms, (2) no increase in the number of viable microorganisms counted from the previous contact time, and (3) the cosmetic formulations meet criterion A or B of the preservative effectiveness test for all the reference microorganisms (Table 6 and Table 7). Among all the tested bioferments, fermented cucumber, celery, and the mixture of white cabbage, cucumber, and celery (1:1:1) fulfilled criterion A of the preservative effectiveness test for *S. aureus*, *E. coli*, and *A. brasiliensis.* Unfortunately, fermented cucumber, celery, and the mixture of white cabbage, cucumber, and celery (1:1:1) are beyond the criterion of the preservative effectiveness test for *P. aeruginosa* and *C. albicans*. In turn, the synthetic preservative fulfilled criterion A of the preservative effectiveness test for *S. aureus* and *P. aeruginosa*, as well as criterion B for *A. brasiliensis*. However, the synthetic preservatives do not fulfill the criterion of the preservative effectiveness test for *E. coli* and *C. albicans*. Fermented cabbage fulfilled criterion A of the preservative effectiveness test only for *A. brasiliensis*. Fermented cucumber, celery, and a mixture of white cabbage, cucumber, and celery (1:1:1) at a 6% concentration have comparable activity with 0.5% sodium benzoate in inhibiting the growth of pathogens in the o/w emulsion. Therefore, both fermented cucumber and celery and a mixture of white cabbage, cucumber, and celery (1:1:1) and sodium benzoate should be used in combination with other preservatives/compounds to ensure full protection of the cosmetic formulation and meet the criterion of the preservative effectiveness test for all reference strains of microorganisms.

## 3. Discussion

The extensive demands of customers for products containing fewer chemicals and more natural bio-preserving products have resulted in extensive research on new antimicrobial compounds that could effectively inhibit the development of pathogens in cosmetic formulations. The FVEs, a waste product from the food industry, are a rich source of antimicrobial compounds, which may act as substitutes for synthetic preservatives or preservative boosters, reducing the concentration of preservatives added to the formulations.

During fermentation processes, LAB produce a wide range of metabolites that constitute an effective weapon in the fight against pathogenic microflora [2]. The FVEs are a rich source of *Lactobaccillus* sp. and *Lactococcus* sp. The range of LAB in bioferments varies between 1 × 10^4^ and 1 × 10^8^ cfu/mL for *Lactobaccillus* sp. and 5 × 10^3^ and 1 × 10^9^ cfu/mL for *Lactococcus* sp., and they tend to have an acidic pH (average pH 3.46 for lyophilizate samples). The amount of LAB in FVEs and their pH are closely related to the fermentation stages. It was found that between 3 and 7 days after the start of white cabbage fermentation, heterofermentative *Leuconostoc* sp. is usually succeeded by the more acid-tolerant homofermentative *Lactobacillus* sp., due to the accumulation of lactic acid to 1% (wt/vol) or more and the decrease in pH below 4.5 [22]. Furthermore, *Lactobacillus plantarum* completes the fermentation of white cabbage with a final pH of approximately 3.5. These results correspond to the results obtained by us and show that fermented white cabbage extract contains much less *Leuconostoc* sp. (6 × 10^3^ cfu/mL) than *Lactobacillus* sp. (2 × 10^7^ cfu/mL), with a final pH 4 in complete fermentation. Furthermore, it is well known that the LAB production of weak organic acids (acetic acid, citric acid, lactic acid, formic, propionic acid) results in an acidic environment, which generally restricts the growth of both bacteria and fungi, including many pathogenic and spoilage microbes [23]. It was shown that the growth of pathogens depends on the pH of the food products [24]. The growth of *S. aureus* and *E. coli* is inhibited when the pH of the food product drops below pH 4. In our studies, the acidic pH of lyophilizates from the fermented cucumber (pH 2.90), fermented celery (pH 3.37), fermented cauliflower (pH 3.48), and fermented white cabbage (pH 3.91) showed antibacterial activity against *S. aureus* and *E. coli*. Unfortunately, this correlation was not visible in the case of all FVEs. It seems that the acidic pH of FVEs is not always sufficient to inhibit the growth of pathogenic bacteria. However, the weak organic acids produced by LAB are only active in their undissociated form, which is capable of crossing the cell membrane of a microorganism [25]. In the cytosol (pH 6–7), the acid transforms into its dissociated form and causes a decrease in the cytosolic pH, resulting in microbial death or the inhibition of growth, causing very extended lag phases. Therefore, the low pH of FVEs may have a beneficial effect on inhibiting the microorganisms’ growth in the o/w formulation.

Vegetables generate a large number of various compounds which may be fermented and metabolized by LAB, leading to the creation of an attractive biologically active raw material for the cosmetics and pharmaceutical industries. The results of the diffusion test showed that fermented white cabbage, cucumber, and celery and their mixture (1:1:1) strongly inhibited the growth of *S. aureus*, *P. aeruginosa*, *E. coli*, and had weak antifungal activity against *C. albicans* and *A. brasiliensis*. Not many studies on fermented plant antimicrobial activity are available. Fermented carrot (*Daucus carota*) with *Lactobacillus* sp. (*L. plantarum*, *L. casei*, *L. paracasei*, *L. rhamnosus*) showed antibacterial activity against food-borne *Listeria monocytogenes, Salmonella* spp., *S. aureus*, *Bacillus cereus*, and weak activity against *E. coli* in the disk diffusion assay [26]. In our study, no antibacterial and antifungal activities for fermented carrots were observed in the disk diffusion test. It seems that scientists focus their interests on the antimicrobial properties of LAB isolated from fermented vegetables, rather than the antimicrobial activity of fermented vegetable extracts. The *Lactobacillus* sp. (*L. paracasei*, *L. casei*, *L. rhamnosus*) isolated from fermented beetroot [27], *L. plantarum*, *L. fermentum*, *L. acidophilus*, and *Leuconostoc mesenteroides* isolated from the spontaneous fermentation of cucumber [28], *Lactobacillus* sp. and *Leuconostoc* sp. from fermented white cabbage [29], *Pediococcus pentosaceus* from fermented garlic [30], and the *L. plantarum*, *L. pentosus*, and *L. fermentum* strains isolated from fermented radish [31] showed antibacterial activity.

The FVEs, due to their antimicrobial activities, may emerge as effective strategies for extending the shelf life of formulations by inhibiting the growth of pathogenic microorganisms. The use of fermented cucumber, celery, and the mixture of white cabbage, cucumber, and celery (1:1:1) in formulation significantly reduced *S. aureus*, *E. coli*, and *A. brasiliensis* growth and fulfilled criterion A of the preservative efficiency test, but they were inactive against *P. aeruginosa* and *C. albicans*. Therefore, the tested FVEs can be valuable raw materials in supporting the preservative system but may not be able to replace synthetic preservatives in o/w formulation. Also, sodium benzoate, categorized as a natural preservative, is permitted for use in cosmetics and personal care products at a maximum concentration 0.5% in leave-on products [32] but does not meet the criterion of the preservative efficiency test for all the tested reference microorganism strains. Sodium benzoate inhibited the growth of *S. aureus*, *P. aeruginosa*, *A. basiliensis*, but was not active against *E. coli* and *C. albicans*. In the case of dermal formulations with high water content, the efficacy of conventional preservatives (especially used in low concentrations) does not always guarantee complete protection against pathogenic microorganisms. It was shown that phenoxyethanol (0.25%) and a combination of methylparaben and propylparaben (7:3, *w*/*w*; 0.1% and 0.4%) in cosmetic emulsions do not fulfill criterion A or B of the preservative efficiency test for *P. aeruginosa* and *C. albicans* [33]. Therefore, the preservative system efficiency should always be confirmed by a challenge test.

Our research data are the first in the scientific literature to attempt to use fermented vegetable extracts as a preservative system in cosmetic products. A similar result was obtained for *Lactobacillus* ferment (mixture of antimicrobial peptides not specified by authors) added at concentrations of 1% and 1.5% to cosmetic emulsions [33]. The *Lactobacillus* ferment at a 1% concentration significantly reduced *S. aureus* and *A. brasiliensis* growth and fulfilled criterion A of the preservative efficiency test but does not protect the emulsion against *P. aeruginosa* and *C. albicans*. The *Lactobacillus* ferment at a concentration of 1.5% meets criterion A of the preservative efficiency test for *S. aureus*, *P. aeruginosa*, *A. brasiliensis* but does not protect the formulation against *C. albicans*. Therefore, the *Lactobacillus* ferment should be used in combination with other preservatives that are efficacious against yeast. Currently available on the cosmetics market are several natural preservatives for cosmetics application based on LAB ferment filtrate, including Leucidal^®^ Liquid (INCI: *Leuconostoc/Radish Root Ferment Filtrate*), AMTicide^®^ Coconut (INCI: *Lactobacillus* and Cocos Nucifera), Leucidal^®^ Liquid Complete (INCI: *Leuconostoc*/Radish Root Ferment Filtrate and Lactobacillus and Cocos Nucifera (Coconut) Fruit Extract), Leucidal^®^ Liquid PT (INCI: *Lactobacillus* Ferment), Leucidal^®^ SF Complete (INCI: *Lactobacillus* Ferment and *Lactobacillus* and Cocos Nucifera (Coconut) Fruit Extract), AMTicide^®^ VAF (*Bacillus* Ferment and *Saccharomyces* Ferment Filtrate), Arborcide^®^ OC (*Leuconostoc* Kimchi Ferment Filtrate) produced by Active Micro Technologies LLC [34]. Leucidal^®^ Liquid was the first fermented bio-preservative on the market, based on antimicrobial peptides synthesized during the fermentation of Daikon radish root (*Raphanus sativus*) by Leuconostoc. Leucidal^®^ Liquid is accepted by ECOCERT as an alternative preservative used in organic cosmetics [35]. According to the manufacturer’s declaration, Leucidal^®^ Liquid inhibits the growth of *S. aureus*, *E. coli*, *P. aeruginosa*, *C. albicans*, *A. niger*, *K. pneumoniae*, and *B. cepacia* bacteria in cosmetic preparations (especially face creams, body lotions, with hydroxy acids, hair conditioners) that do not require production temperatures of higher than 70 °C, has stability in a wide pH range from 3 to 8, and its recommended concentration in cosmetic formulas is 2–4% [36]. In addition to its preservative activities, Leucidal^®^ Liquid increases the level of skin hydration and antioxidant capacity (0.001%) comparable to 200 μM Trolox. Li et al. [37] showed that the antimicrobial activity of commercial fermented radish kimchi is attributed to salicylic acid against Gram-negative bacteria, as well as didecyldimethylammonium salt against Gram-positive activity. Unfortunately, during the analysis, it was unable to detect antimicrobial peptides in the studied samples of the commercial fermented radish kimchi [37]. It was also showed that the high concentration of Leucidal^®^ Liquid exerted antibacterial activity (bacteriostatic, not bactericidal) against *S. aureus*, *Salmonella enterica*, *P. aeruginosa*, *E. coli*, and *Enterococcus faecalis*, but this activity poses low selectivity and may be problematic when in contact with the skin microbiome [38].

FVEs are rich sources of antioxidants (ascorbic acid, polyphenols) that may act as preservative boosters in topical formulations by preventing the oxidation of a product [39]. All the tested FVEs showed antioxidant activities. The most significant antioxidant properties were shown in fermented red cabbage, radish, and beetroot, while fermented garlic poses weaker radical scavenging activity. The literature data confirm the results obtained by us. Generally, fermented red cabbage [40,41], fermented white cabbage (sauerkraut) [42], fermented red radish [43], fermented beetroot [44], fermented garlic [45], fermented carrot juice [46], fermented cauliflower [47] have weaker antioxidant activity compared to fresh vegetables, and this difference is associated with the decreasing number of polyphenols during the fermentation process. Moreover, compounds with antioxidant activity are always present in cosmetic products as active agents for the prevention of hyperpigmentation and slowing down of the skin-aging process [48,49]. The FVEs with significant antioxidant properties may act as biologically active raw materials in anti-aging formulations as well as preservative boosters in cosmetic and pharmaceutical products.

## 4. Materials and Methods

### 4.1. Microorganisms

*Pseudomonas aeruginosa* ATCC 9027, *Escherichia coli ATCC* 8739, *Staphylococcus aureus* ATCC 6538, *Candida albicans* ATCC 10,231 and *Aspergillus brasiliensis* ATCC 16,404 were used. The microorganisms were activated through double passaging as follows: bacteria on TSA medium (Trypticase Soy Agar; BioMerieux, Craponne, France) (37 °C, 24 h), yeast on SDA medium (Sabouraud Dextrose Agar; BioMerieux, Craponne, France) (25 °C, 48 h), and mold on SDA medium (BioMerieux, France) (25 °C, 5 days).

### 4.2. Fermented Vegetable Extracts (FVEs)

Fermented cauliflower (cauliflower, garlic, dill weed, water, salt), radish (radish, water, salt), carrot (carrot, water, salt), white cabbage (white cabbage, salt), red cabbage (red cabbage, salt), garlic (garlic, water, dill weed, salt), cucumber (cucumber, garlic, dill weed, water, salt), beetroot (beetroot, water, salt), and celery (celery, water, salt) were purchased from an ecological farm located in the area of the Barycz Valley Landscape Park, Poland. All fermented vegetables are unpasteurized products.

The FVEs were collected from the vegetables and 100 μL of each bioferment was spread onto MRS agar (*Lactobacilli* growth), M17 agar (*Lactococcus* growth), and MRS agar supplemented with 0.05% cysteine hydrochloride (*Bifidobacterium* growth) for the determination of the amount of LAB in the fermented vegetable extracts. The plates for *Lactobacilli* and *Lactococcus* growth were incubated at 37 °C for 48 h, while the plates for *Bifidobacterium* sp. were incubated under anaerobic conditions for 72 h at 37 °C and the colonies that formed on plates were counted. *Lactobacillus rhamnosus GG* (ATCC 53103), *Lactococcus lactis* (ATCC 11454), and *Bifidobacterium animalis* BB-12 as probiotic supplements were positive controls. Also, pH measurements of the bioferments before and after lyophilization were carried out. The yield rates of lyophilization of the FVEs are shown in Table 8.

The samples were stored at −80 °C until the analysis.

### 4.3. Determination of the Antioxidant Activity of FVEs—ABTS Assay

The ABTS (2,2′-azinobis-(3-ethylbenzthiazolin-6-sulfonic acid)) assay measures the ability of an antioxidant to stabilize the ABTS radical cation (ABTS^+^). The ABTS^·+^ is a green-blue chromophore produced through a reaction between 7 mM aqueous ABTS and 2.45 mM potassium persulfate (K_2_S_2_O_8_) mixed in a ratio of 1:1 and incubated overnight (12–16 h) at room temperature in the dark. The ABTS solution was diluted to obtain 0.7 absorbance at 734 nm. In a 96-well microtiter plate, a 10 μL sample of the FVEs or ascorbic acid as a positive control (both at the concentration range of 0.015–250 mg/mL obtained by a two-fold dilution in water) was mixed with 190 μL of ABTS radical solution and incubated for 30 min at room temperature in the dark. The blanc well was obtained by mixing 10 μL of water and 190 μL of the ABTS radical solution. For each test, three replicates were performed. The sample and blanc absorbances were determined at a 734 nm wavelength in a microplate reader (Synergy H4, BioTek, Agilent, Santa Clara, CA, USA).

The radical scavenging activity (RSA) of the fermented vegetable extract was calculated using the following formula:

% RSA = ((Abs _blanc_ − Abs _sample_)/Abs _blanc_) × 100

where:

% RSA—percent of radical scavenging activity

Abs _blanc_—absorbance of ABTS

Abs _sample_—absorbance of a sample

The antiradical activity of a fermented plant extract was also expressed as EC50 (mg/mL), the concentration of sample required to cause 50% ABTS inhibition. The EC50 value was calculated by a graphical method as the effective concentration that results in 50% inhibition of radical formation.

### 4.4. Determination of the Antimicrobial Activity of FVEs and Their Mixture

The antibacterial and antifungal activity of FVEs as well as inoculum preparation were evaluated using the methods described by EUCAST [50,51,52]. Several colonies of overnight cultures for bacteria and 48 h for yeast were suspended in saline to obtain a density equal to the McFarland turbidity standard of 0.5 (approximate cell density of 1.5 × 10^8^ CFU/mL for bacteria, and 1.5 × 10^6^ CFU/mL for yeast). Sporulated *A. brasiliensis* colonies were covered with 5 mL of sterile water supplemented with 0.1% Tween 20. Then, the conidia were rubbed with a sterile cotton swab and transferred with a pipette to a sterile tube. The suspension was homogenized for 15 s with a vortex mixer. The suspension was then checked for the presence of hyphae or clumps via a cell-counting hematocytometer chamber. If a significant number of hyphae were detected, 5 mL of the suspension was transferred to a sterile syringe attached to a sterile filter with a pore diameter of 11 μm, filtered and then collected in a sterile tube. This step removes the hyphae and yields a suspension composed of conidia. If many clumps were detected, the inoculum was shaken again in a vortex mixer for a further 15 s. This step was repeated until clumps were no longer encountered. The suspension of *A. brasiliensis* conidia was adjusted with sterile distilled water to the McFarland turbidity standard of 0.5 (approximate cell density of 1.5 × 10^6^ CFU/mL). Suspensions of microorganisms were spread over the TSA and SDA agar plates (BioMerieux, Craponne, France), respectively, using sterile cotton swabs. Then, 10 µL of fermented vegetable lyophilizates (1 g/mL) and their mixtures (1:1) were placed on the agar surface. Gentamycin (120 μg) and nystatin (100 IU) (BTL, Warsaw, Poland) were used as controls. All bacterial plates were incubated at 37 °C for 24 h and fungal plates at 25 °C for 48 h (*C. albicans*) and 5 days (*A. brasiliensis*), respectively. The diameter of the zone of inhibition was measured in mm. All tests were conducted in triplicate and data from experiments were calculated as mean ± SD.

### 4.5. Preparation of o/w Emulsions

The composition of the formulations is shown in Table 9. Glyceryl Stearate (and) Ceteareth-20 (and) Ceteareth-12 (and) Cetearyl Alcohol (and) Cetyl Palmitate (Emulgade^®^ SE-PF, BASF Poland), Cetearyl Isononanoate (Cetiol^®^ SN, BASF, Warszawa, Poland), Octyldodecanol (Eutanol^®^ G, BASF, Poland), Paraffinum Liquidum (Avena, Kraków, Poland), Cetyl Alcohol (Lanette^®^ 16, BASF, Poland), Dimethicone (BASF, Poland) as oil phase, and Glycerin (POCH S.A, Poland), water as aqueous phase were homogenized for 3 min using the Heidolph SilentCrusher M homogenizer (Heidolph Instruments GmbH & Co. KG, Schwabach, Germany) at approximately 15,000 rpm. Then, the emulsion was gently stirred using the blender RW 16 (IKA^®^ Werke GmbH & Co. KG, Staufen im Breisgau, Germany), and the FVE lyophilizates and their mixtures (1:1:1) were added at a concentration of 6%. A preservative of natural origin (sodium benzoate, Pol-Aura, Olsztyn, Poland) was added at 0.5% concentration as the maximum permissible concentration in cosmetics. The emulsion without preservatives and FVEs was a reference sample.

### 4.6. Determination of Microbiological Purity of the o/w Emulsions

The enumeration and detection of aerobic mesophilic bacteria, yeast, and mold present in the emulsion were performed according to PN-EN ISO 21149:2017-07/A1:2023-01 [15] and PN-EN ISO 16212:2017-08/A1:2023-01 [20], respectively. Detection of *S. aureus*, *P. aeruginosa*, *E. coli*, and *C. albicans* in emulsions were performed according to the PN-EN ISO 22718:2016-01/A1:2023-01 [16], PN-EN ISO 22717:2016-01/A1:2023-03 [17], PN-EN ISO 21150:2016-01/A1:2023-03 [18], and PN-EN ISO 18416:2016-01/A1:2023-03 [19]. The 1 g o/w emulsion with FVEs/preservative/control (without FVEs or preservative) was mixed with 9 mL of Eugon LT 100 broth medium to neutralize the possible preservative effect. Then, 1 mL of these samples were grown on selective agar such as Cetrimide, Sabouraud Dextrose with Chloramphenicol, Baird-Parker, and MacConkey for *P. aeruginosa*, *C. albicans*, *S. aureus*, and *E. coli*, respectively. The agar plates were incubated 24 h (bacteria), 48 h (yeast), and 5 days (mold) at 37 °C and 25 °C, respectively.

### 4.7. Determination of the Preservation Efficacy (Challenge Test) of the o/w Emulsions

Evaluation of the antimicrobial protection of a cosmetic product was performed according to PN-EN ISO 11930:2019-03/A1:2023-02 [21]. Inoculation of the test microorganism suspensions was prepared by the addition of 0.02 mL of calibrated inoculum (each strain separately) to 20 g emulsion o/w sample to obtain a final bacterial concentration of between 1 × 10^5^ and 1 × 10^6^ CFU/mL and fungi of between 1 × 10^4^ and 1 × 10^5^ CFU/mL in the formulation. The inoculated containers were mixed thoroughly and incubated in the dark at 20 °C—25 °C. The number of viable microorganisms in the formulations was determined by the plate count method at the proper times, 0, 7, 14, and 28 days after inoculation for bacteria and yeast, and 0, 14, and 28 days for molds. A sample of 1 g of emulsion was then transferred to 9 mL of Eugon LT 100 broth (Graso Biotech, Owidz, Poland) and pre-incubated for 30 min at room temperature; a 10-fold dilution method was carried out. Triplicate plating of each dilution was performed with TSA agar for bacteria, SDA agar for yeast, and PDA agar for mold. The plates were incubated at 37 °C for 24 h (bacteria), 48 h (*C. albicans*), and 30 °C for 5 days (*A. brasiliensis*), respectively. The CFU per plate (30–300 colonies for bacteria and *C. albicans*, 15–150 colonies for *A. brasiliensis*) were counted to determine the number of surviving microorganisms per gram of tested cosmetic product. The results were expressed as a log reduction value (log CFU/g).

R = log_0_ − log_t_

where:

R—logarithmic reduction in the number of viable microorganisms

log_0_—logarithm of the number of microorganisms in 1 g of cosmetic immediately after infection

log_t_—logarithm of the number of microorganisms in 1 g of cosmetic after a specified time (7, 14, 28 days) after infection

For each time and each strain, the log reduction value is calculated and compared to the minimum values required for evaluation criteria A or B of the preservation efficacy test (Annex B in PN-EN ISO 11930:2019) [21]. The formulation meets Criterion A when the cosmetic product is protected against microbial proliferation that may present a potential risk for the users and the cosmetic product is deemed to have met the requirements of the standard without any additional rationale (recommended efficacy of antimicrobial preservation for topical preparations). The formulation meets Criterion B when the microbiological risk analysis demonstrates the existence of control factors not related to the formulation (e.g., a protective package such as pomp provides a higher level of protection than a jar), indicating that the microbiological risk is tolerable. All tests were conducted in triplicate and data from the experiments were calculated as mean ± SD. The standard deviation for the test of microorganism population viability does not exceed 0.5 logarithmic unit.

### 4.8. Statistical Analysis

All tests were conducted in triplicate and data from experiments were calculated as mean ± SD. The standard deviation for the test of microorganism population viability does not exceed 0.5 logarithmic unit.

## 5. Conclusions

The food industry generates a large amount of waste whose potential remains underestimated and among them are FVEs. Fermentation products are rich sources of biologically active compounds, emphasizing their use as natural preservatives in cosmetic and pharmaceutical products. Fermented cucumber, fermented celery, and a mixture of fermented white cabbage, cucumber, and celery (1:1:1) can be valuable raw materials supporting the preservative system, which can significantly reduce the concentration of synthetic preservatives used in formulations. Finding a suitable type of preservative or preservative system to incorporate into the specific formula, which satisfies all preservation and toxicological safety criteria, presents a challenge for the cosmetic microbiologists. Further, to ensure the safety of fermented vegetables, the bacterial strains used for transforming the components of plant products into active entities must be isolated and their safety should be verified. Another important issue is determining the chemical composition of fermented vegetable extracts to find out the molecular components responsible for the antimicrobial activity and to highlight their mechanism of action. The final stage should include a preservative effectiveness test for improvements in the effectiveness of the compounds isolated from FVEs as a preservative system to inhibit the growth of microorganisms in the formulation during manufacturing, storage, and safety use by consumers. Therefore, future studies should be directed toward characterizing the bacterial strains responsible for fermentation, determining the composition of bioferments, isolating active compounds with antimicrobial properties from fermentation wort, and confirming their use as substitutes for preservatives in cosmetic products.

## Figures and Tables

**Table 1:** The number of lactic acid bacteria in the fermented vegetable extracts [cfu/mL].

Fermented Vegetables/Probiotics	[cfu/mL]		
	*Lactobacillus* sp.	*Lactococcus* sp.	*Bifidobacterium* sp.
cauliflower	1 ± 0.24 × 10^6^	8 ± 1.47 × 10^7^	-
radish	1 ± 0.19 × 10^7^	4 ± 1.31 × 10^7^	-
carrot	6 ± 1.23 × 10^7^	1 ± 0.33 × 10^9^	-
white cabbage	2 ± 3.26 × 10^7^	6 ± 0.13 × 10^3^	-
red cabbage	2 ± 0.0 × 10^5^	3 ± 0.09 × 10^5^	-
garlic	1 ± 0.22 × 10^8^	7 ± 2.84 × 10^7^	-
cucumber	8 ± 1.42 × 10^7^	2 ± 1.36 × 10^7^	-
beetroot	1 ± 0.27 × 10^4^	5 ± 0.41 × 10^3^	-
celery	2 ± 1.63 × 10^5^	1 ± 0.22 × 10^4^	-
*Lactobacillus rhamnosus*	1 ± 0.31 × 10^9^	-	-
*Lactococcus lactis*	-	9 ± 0.18 × 10^8^	-
*Bifidobacterium animalis*	-	-	2 ± 0.00 × 10^9^

**Table 2:** The pH value of fermented vegetable extracts before (pH 1) and after lyophilization (pH 2).

FVEs	pH 1	pH 2
cauliflower	4.44 ± 0.0	3.48 ± 0.0
radish	4.43 ± 0.01	3.81 ± 0.0
carrot	3.42 ± 0.0	3.24 ± 0.01
white cabbage	4.00 ± 0.0	3.91 ± 0.01
red cabbage	4.21 ± 0.0	3.47 ± 0.0
garlic	4.22 ± 0.01	3.52 ± 0.01
cucumber	4.00 ± 0.0	2.90 ± 0.0
beetroot	4.11 ± 0.0	3.46 ± 0.0
celery	4.37 ± 0.01	3.37 ± 0.0

**Table 3:** Radical scavenging activity of fermented vegetable extracts.

Fermented Vegetables	EC50 [mg/mL]
cauliflower	76.21
radish	16.83
carrot	265.16
white cabbage	36.39
red cabbage	7.14
garlic	201.50
cucumber	169.67
beetroot	17.58
celery	140.83
ascorbic acid	0.10

**Table 4:** Antibacterial and antifungal activity of fermented vegetable extracts and their mixtures (10 μL) in agar diffusion method, diameter inhibition zone (mm).

Fermented Vegetables	Microorganism				
	*S. aureus*	*P. aeruginosa*	*E. coli*	*C. albicans*	*A. brasiliensis*
cauliflower	4 ± 0.62	6 ± 0.73	4 ± 0.50	0 ± 0.00	0 ± 0.00
radish	0 ± 0.00	0 ± 0.00	0 ± 0.00	0 ± 0.00	0 ± 0.00
carrot	0 ± 0.00	6 ± 0.00	0 ± 0.00	0 ± 0.00	0 ± 0.00
white cabbage	10 ± 0.43	11 ± 0.33	8 ± 0.42	5 ± 0.55	0 ± 0.00
red cabbage	0 ± 0.00	6 ± 0.54	0 ± 0.00	0 ± 0.00	0 ± 0.00
garlic	0 ± 0.00	5 ± 0.00	0 ± 0.00	0 ± 0.00	0 ± 0.00
cucumber	9 ± 0.43	9 ± 0.43	10 ± 0.44	5 ± 0.00	0 ± 0.00
beetroot	0 ± 0.00	0 ± 0.00	0 ± 0.00	0 ± 0.00	0 ± 0.00
celery	13 ± 0.42	13 ± 0.52	10 ± 0.41	0 ± 0.00	3 ± 0.02
cauliflower + white cabbage	0 ± 0.00	7 ± 0.00	0 ± 0.00	4 ± 0.51	0 ± 0.00
cauliflower + red cabbage	0 ± 0.00	9 ± 0.50	0 ± 0.00	0 ± 0.00	0 ± 0.00
cauliflower + cucumber	0 ± 0.00	3 ± 0.00	0 ± 0.00	0 ± 0.00	0 ± 0.00
cauliflower + celery	0 ± 0.00	10 ± 0.52	0 ± 0.00	0 ± 0.00	0 ± 0.00
celery + white cabbage	0 ± 0.00	6 ± 0.51	0 ± 0.00	0 ± 0.00	0 ± 0.00
celery + red cabbage	0 ± 0.00	8 ± 0.00	0 ± 0.00	7 ± 0.00	0 ± 0.00
celery + cucumber	0 ± 0.00	5 ± 0.00	4 ± 0.05	0 ± 0.00	0 ± 0.00
cucumber+ white cabbage	0 ± 0.00	7 ± 0.50	0 ± 0.00	5 ± 0.03	0 ± 0.00
red cabbage + cucumber	0 ± 0.00	8 ± 0.52	0 ± 0.00	5 ± 0.54	0 ± 0.00
red cabbage + white cabbage	0 ± 0.00	7 ± 0.52	0 ± 0.00	5 ± 0.00	0 ± 0.00
white cabbage + cucumber + cauliflower	7 ± 0.39	7 ± 0.53	5 ± 0.53	0 ± 0.00	0 ± 0.00
white cabbage + cucumber + celery	8 ± 0.00	10 ± 0.00	6 ± 0.50	5 ± 0.54	0 ± 0.00
white cabbage + cucumber + cauliflower + celery	8 ± 0.44	8 ± 0.50	6 ± 0.00	0 ± 0.00	0 ± 0.00
gentamycin	30 ± 0.44	31 ± 0.43	32 ± 0.41	-	-
nystatin	-	-	-	14 ± 0.45	13 ± 0.44

**Table 5:** Microbiological purity of o/w emulsion (base), o/w emulsion with preservative, or fermented vegetable extracts, according to PN-EN ISO standards.

	Base	Preservative	White Cabbage	Cucumber	Celery	White Cabbage + Cucumber + Celery (1:1:1)	Ref.
Total aerobic mesophilic microorganisms	<100 cfu/g	<100 cfu/g	<100 cfu/g	<100 cfu/g	<100 cfu/g	<100 cfu/g	[15]
*Staphylococcus aureus*	negative	negative	negative	negative	negative	negative	[16]
*Pseudomonas aeruginosa*	negative	negative	negative	negative	negative	negative	[17]
*Escherichia coli*	negative	negative	negative	negative	negative	negative	[18]
*Candida albicans*	negative	negative	negative	negative	negative	negative	[19]
Mold–Yeast	0	0	0	0	0	0	[20]

**Table 6:** Evaluation criteria for the preservation efficacy test for o/w emulsions with preservatives and fermented vegetable extracts according to PN-EN ISO 11930:2019-03/A1:2023-02 standard (Annex B) [21].

	Logarithm of Reduction Values of Viable Cells																	
	*S. aureus*					*P. aeruginosa*				*E. coli*			*C. albicans*			*A. brasiliensis*		
Time [days]	7	14	28	7	14		28	7	14		28	7		14	28		14	28
emulsion o/w																		
Base	<3	≥3 and NI	<3	<3	↑		<3	↑	↑		↑	↑		↑	↑		≥0 *	≥0 *
Preservative	≥3 and NI	≥3 and NI	≥3 and NI	≥3 and NI	≥3 and NI		≥3 and NI	<3 and NI	<3 and NI		<3 and NI	↑		↑	↑		≥0 *	≥0 *
White cabbage	<3	≥3 and NI	↑	≥3 and NI	≥3 and NI		<3	≥3 and NI	<3		↑	↑		↑	↑		≥1 and NI	≥1 and NI
Cucumber	≥3 and NI	≥3 and NI	≥3 and NI	≥3 and NI	≥3		<3	≥3 and NI	≥3 and NI		≥3 and NI	↑		↑	↑		≥1 and NI	≥1 and NI
Celery	≥3 and NI	≥3 and NI	≥3 and NI	≥3 and NI	≥3		<3	≥3 and NI	≥3 and NI		≥3 and NI	↑		↑	<1		≥0 *	≥1 and NI
White cabbage + Cucumber + Celery 1:1:1	≥3 and NI	≥3 and NI	≥3 and NI	≥3 and NI	<3		↑	≥3 and NI	≥3 and NI		≥3 and NI	↑		↑	↑		≥0 *	≥1 and NI

Legends: ↑—growth of microorganisms above initial level of contamination; NI—did not increase in the count from the previous contact time; *—the number of microorganisms does not increase in relation to the initial level of contamination of the sample.

**Table 7:** Criteria of preservation efficacy test for o/w emulsion with preservatives and fermented vegetable extracts according to PN-EN ISO 11930:2019-03/A1:2023-02 standard [21].

o/w Emulsion	*S. aureus*	*P. aeruginosa*	*E. coli*	*C. albicans*	*A. brasiliensis*
Base	-*	-	-	-	B
Preservative	A	A	-	-	B
White cabbage	-	-	-	-	A
Cucumber	A	-	A	-	A
Celery	A	-	A	-	A
White cabbage + Cucucmber + Celery(1:1:1)	A	-	A	-	A

Legends: * beyond the criterion; A—recommended efficacy of antimicrobial preservation for topical preparations; B—microbiological risk for topical preparations is tolerable.

**Table 8:** The yield rates of lyophilization of the FVEs [%].

FVEs	Lyophilization Efficiency [%]
cauliflower	2.3
radish	3.1
carrot	5.2
white cabbage	11.9
red cabbage	4.7
garlic	3.2
cucumber	6.5
beetroot	6.1
celery	4.8

**Table 9:** Composition of the emulsions: E1—emulsion without preservative/fermented vegetable extracts; E2—emulsion with preservative; E3—emulsion with fermented vegetable extracts.

Ingredient *	Percentage by Weight		
	E1	E2	E3
Cetyl Alcohol	2	2	2
Glyceryl Stearate (and) Ceteareth-20 (and) Ceteareth-12 (and) Cetearyl Alcohol (and) Cetyl Palmitate	8	8	8
Paraffinum Liquidum	3	3	3
Dimethicone	0.5	0.5	0.5
Octyldodecanol	3	3	3
Cetearyl Isononanoate	3	3	3
Glycerin	3	3	3
Sodium benzoate	0	0.5	0
Fermented vegetable extract	0	0	6
Aqua	77.5	77	71.5

* INCI Name.

## Data Availability

Data is contained within the article.

## References

[1] Mora-Villalobos J.A., Montero-Zamora J., Barboza N., Rojas-Garbanzo C., Usaga J., Redondo-Solano M., Schroedter L., Olszewska-Widdrat A., López-Gómez J.P. (2020). Multi-product lactic acid bacteria fermentations: A review. Fermentation.

[2] Saranraj P., Naidu M.A., Sivasakthivelan P. (2013). Lactic acid bacteria and its antimicrobial properties: A review. Int. J. Pharm. Biol. Arch..

[3] Şanlıbaba P., Güçer Y. (2015). Antimicrobial activity of lactic acid bacteria. J. Int. Sci..

[4] Erdem Büyükkiraz M., Kesmen Z. (2022). Antimicrobial peptides (AMPs): A promising class of antimicrobial compounds. J. Appl. Microbiol..

[5] Savard T., Beaulieu C., Gardner N.J., Champagne C.P. (2002). Characterization of spoilage yeasts isolated from fermented vegetables and inhibition by lactic, acetic and propionic acids. Food Microbiol..

[6] Peláez A.L., Cataño C.S., Yepes E.Q., Villarroel R.G., De Antoni G.L., Giannuzzi L. (2012). Inhibitory activity of lactic and acetic acid on Aspergillus flavus growth for food preservation. Food Control.

[7] Ray B. (2019). Diacetyl of lactic acid bacteria as a food biopreservative. Food Biopreservatives of Microbial Origin.

[8] Alvarez-Sieiro P., Montalbán-López M., Mu D., Kuipers O.P. (2016). Bacteriocins of lactic acid bacteria: Extending the family. Appl. Microbiol. Biotechnol..

[9] Rodali V.P., Lingala V.K., Karlapudi A.P., Indira M., Venkateswarulu T.C., John Babu D. (2013). Biosynthesis and potential application of bacteriocins. J. Pure Appl. Microbiol..

[10] Kumariya R., Garsa A.K., Rajput Y.S., Sood S.K., Akhtar N., Patel S. (2019). Bacteriocins: Classification, synthesis, mechanism of action and resistance development in food spoilage causing bacteria. Microb. Pathog..

[11] Rai M., Pandit R., Gaikwad S., Kövics G. (2016). Antimicrobial peptides as natural bio-preservative to enhance the shelf-life of food. J. Food Sci. Technol..

[12] Mathur H., Field D., Rea M.C., Cotter P.D., Hill C., Ross R.P. (2017). Bacteriocin-antimicrobial synergy: A medical and food perspective. Front. Microbiol..

[13] Yue L., Song L., Zhu S., Fu X., Li X., He C., Li J. (2024). Machine learning assisted rational design of antimicrobial peptides based on human endogenous proteins and their applications for cosmetic preservative system optimization. Sci. Rep..

[14] Herman A., Herman A.P. (2023). Biological activity of fermented plant extracts for potential dermal applications. Pharmaceutics.

[15] (2023). Cosmetics-Microbiology–Enumeration and Detection of Aerobic Mesophilic Bacteria.

[16] (2023). Cosmetics-Microbiology–Detection of Staphylococcus aureus.

[17] (2023). Cosmetics-Microbiology–Detection of Pseudomonas aeruginosa.

[18] (2023). Cosmetics-Microbiology–Detection of Escherichia coli.

[19] (2023). Cosmetics-Microbiology–Detection of Candida albicans.

[20] (2023). Cosmetics-Microbiology–Enumeration of Yeasts and Molds.

[21] (2023). Cosmetics-Microbiology–Evaluation of the Effectiveness of Antimicrobial Protection of a Cosmetic Product.

[22] Cvetković B., Bardić Ž., Jokanović M., Mastilović J. (2008). Technological quality of biofermented white cabbage, cultivar Futoški. Food Feed Res..

[23] Batish V.K., Roy U., Lal R., Grower S. (1997). Antifungal attributes of lactic acid bacteria—A review. Crit. Rev. Biotechnol..

[24] (2010). National Advisory Committee on Microbiological Criteria for Foods. Parameters for determining inoculated pack/challenge study protocols. J. Food Prot..

[25] Hazan R., Levine A., Abeliovich H. (2004). Benzoic acid, a weak organic acid food preservative, exerts specific effects on intracellular membrane trafficking pathways in Saccharomyces cerevisiae. Appl. Environ. Microbiol..

[26] Ricci A., Bernini V., Maoloni A., Cirlini M., Galaverna G., Neviani E., Lazzi C. (2019). Vegetable by-product lacto-fermentation as a new source of antimicrobial compounds. Microorganisms.

[27] Kumari V.B., Huligere S.S., Ramu R., Naik Bajpe S., Sreenivasa M.Y., Silina E., Stupin V., Achar R.R. (2022). Evaluation of probiotic and antidiabetic attributes of lactobacillus strains isolated from fermented beetroot. Front. Microbiol..

[28] Wakil S.M., Laba S.A., Fasika S.A. (2014). Isolation and identification of antimicrobial-producing lactic acid bacteria from fermented cucumber. Afr. J. Biotechnol..

[29] Touret T., Oliveira M., Semedo-Lemsaddek T. (2018). Putative probiotic lactic acid bacteria isolated from sauerkraut fermentations. PLoS ONE.

[30] Ham J.S., Lee S.G., Kim M.K., Oh M.H., Jeong S.G., Kim D.H., Lee S.H., Chae J.E., Lee J.Y., Kang D.K. (2010). Inhibitory activity of garlic fermented by Pediococcus pentosaceus KACC 91419 against antibiotic-resistant pathogens. Asian-Australas. J. Anim. Sci..

[31] Damodharan K., Palaniyandi S., Yang S., Suh J. (2015). In vitro probiotic characterization of Lactobacillus strains from fermented radish and their anti-adherence activity against enteric pathogens. Canad. J. Microbiol..

[32] Annex VI, European Commission Regulation (EC) No 1223/2009 of the European Parliament and of the Council of 30 November 2009 on Cosmetic Products.

[33] Kočevar Glavač N., Lunder M. (2018). Preservative efficacy of selected antimicrobials of natural origin in a cosmetic emulsion. Int. J. Cosmet. Sci..

[34] Active Micro Technologies Products. https://activemicrotechnologies.com.

[35] Herman A. (2019). Antimicrobial ingredients as preservative booster and components of self-preserving cosmetic products. Curr. Microbiol..

[36] Technical Dossier Leucidal^®^ Liquid, Active Micro Technologies. https://activemicrotechnologies.com/?product=leucidal-liquid.

[37] Li J., Chaytor J.L., Findlay B., McMullen L.M., Smith D.C., Vederas J.C. (2015). Identification of didecyldimethylammonium salts and salicylic acid as antimicrobial compounds in commercial fermented radish kimchi. J. Agric. Food Chem..

[38] Wyżga B., Skóra M., Hąc-Wydro K. (2023). The influence of Leucidal–eco-preservative from radish–on model lipid membranes and selected pathogenic bacteria. Chem. Physic. Lipids..

[39] Soto M.L., Parada M., Falqué E., Dominguez H. (2018). Personal-care products formulated with natural antioxidant extracts. Cosmetics.

[40] Wiczkowski W., Szawara-Nowak D., Topolska J. (2015). Changes in the content and composition of anthocyanins in red cabbage and its antioxidant capacity during fermentation, storage and stewing. Food Chem..

[41] Hunaefi D., Akumo D.N., Smetanska I. (2013). Effect of fermentation on antioxidant properties of red cabbages. Food Biotechnol..

[42] Özer C., Yıldırım H.K. (2019). Some special properties of fermented products with cabbage origin: Pickled cabbage, sauerkraut and kimchi. Turk. J. Agric. Food Sci. Technol..

[43] Jing P., Song L.H., Shen S.Q., Zhao S.J., Pang J., Qian B.J. (2014). Characterization of phytochemicals and antioxidant activities of red radish brines during lactic acid fermentation. Molecules.

[44] Sawicki T., Wiczkowski W. (2018). The effects of boiling and fermentation on betalain profiles and antioxidant capacities of red beetroot products. Food Chem..

[45] Tahir Z., Saeed F., Nosheen F., Ahmed A., Anjum F.M. (2022). Comparative study of nutritional properties and antioxidant activity of raw and fermented (black) garlic. Int. J. Food Prop..

[46] Zhang X., Duan W., Zou J., Zhou H., Liu C., Yang H. (2019). Flavor and antioxidant activity improvement of carrot juice by fermentation with Lactobacillus plantarum WZ-01. J. Food Meas. Charact..

[47] Aksay S., Arslan R., Tokbaş H., Eroğlu E.Ç. (2022). Changes in some biochemical properties of Brassica vegetables (Cabbage, Cauliflower and Broccoli) pickles and brines. J. Raw Mater. Process. Foods.

[48] Papaccio F., D′ Arino A., Caputo S., Bellei B. (2022). Focus on the contribution of oxidative stress in skin aging. Antioxidants.

[49] Oresajo C., Pillai S., Manco M., Yatskayer M., McDaniel D. (2012). Antioxidants and the skin: Understanding formulation and efficacy. Dermatol. Ther..

[50] EUCAST Disk Diffusion Method for Antimicrobial Susceptibility Testing. Version 11.0 Valid from January 2023. www.eucast.org.

[51] EUCAST Method for Susceptibility Testing of Yeasts. Version 7.4 Valid from October 2023. www.eucast.org.

[52] EUCAST Method for Susceptibility Testing of Moulds. Version 9.4 Valid from April 2022. www.eucast.org.

